# Water stress resilient cereal crops: Lessons from wild relatives

**DOI:** 10.1111/jipb.13222

**Published:** 2022-02-26

**Authors:** Justine M. Toulotte, Chrysoula K. Pantazopoulou, Maria Angelica Sanclemente, Laurentius A. C. J. Voesenek, Rashmi Sasidharan

**Affiliations:** ^1^ Department of Biology, Plant Ecophysiology, Institute of Environmental Biology Utrecht University Utrecht 3584 CH The Netherlands

**Keywords:** abiotic stress, cereal crops, climate change, crop wild relatives, drought, flooding, grass, water stress

## Abstract

Cereal crops are significant contributors to global diets. As climate change disrupts weather patterns and wreaks havoc on crops, the need for generating stress‐resilient, high‐yielding varieties is more urgent than ever. One extremely promising avenue in this regard is to exploit the tremendous genetic diversity expressed by the wild ancestors of current day crop species. These crop wild relatives thrive in a range of environments and accordingly often harbor an array of traits that allow them to do so. The identification and introgression of these traits into our staple cereal crops can lessen yield losses in stressful environments. In the last decades, a surge in extreme drought and flooding events have severely impacted cereal crop production. Climate models predict a persistence of this trend, thus reinforcing the need for research on water stress resilience. Here we review: (i) how water stress (drought and flooding) impacts crop performance; and (ii) how identification of tolerance traits and mechanisms from wild relatives of the main cereal crops, that is, rice, maize, wheat, and barley, can lead to improved survival and sustained yields in these crops under water stress conditions.

## Graphical abstract

This review provides an overview of drought and flooding resilience mechanisms in major cereal crops uncovered by studying their wild relatives, making a case for wider investigation of crop wild relatives to pave the path for breeding climate‐resilient food crops.

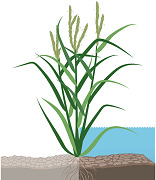

## INTRODUCTION

The increasingly unpredictable and frequent occurrence of severe weather events linked to climate change is devastating food production. Globally, 50%–70% of crop yield reduction is attributed to abiotic stresses (Francini and Sebastiani, 2019) such as heat waves and water extremes (i.e., floods and droughts). The recurrence of these events has been associated with a rise in global temperatures and a heightened variability in precipitation (Hirabayashi et al., 2008). This poses a considerable threat to agricultural productivity world‐wide since most crops are sensitive to extreme environmental conditions. For instance, severe droughts between 1964 and 2007 reduced global production of several cereal crops by 10% (Lesk et al., 2016). In addition, agriculture expansion into flood‐prone areas is expected to further inflate annual crop losses. Understanding mechanisms for abiotic stress resilience in primary crops is thus essential to ensure food security in the near future.

Cereal crops such as maize, wheat, rice, barley, and millet represent a major source of food and caloric intake in several regions of the world. These species account for approximately 50% of proteins and 56% of food energy consumed on earth (Cordain, 1999), providing both dietary energy and feed for meat‐producing animals. Nevertheless, despite their nutritional and economic importance, cereal crops remain sensitive to climate change since they lack adaptive traits for weather extremes. Currently, droughts affect nearly 23 million hectares of rainfed rice reducing global production by 18 million tons a year (O'Toole, 2004; Serraj et al., 2011). Moreover, it is estimated that large‐scale droughts will reduce global yields of wheat (*Triticum aestivum* L.) and maize (*Zea mays* L.) by 21% and 40% (Daryanto et al., 2016).

Modern cultivars of domesticated cereals are less tolerant to environmental stresses than their wild relatives. Initial breeding programs during the green revolution focused primarily on yield, while traits for abiotic stress resilience were neglected. Consequently, several traits linked to stress resistance present in ancestral races were lost in the new cultivars (Portis et al., 2004; Reif et al., 2005; Budak et al., 2015; Hussain, 2015) This reduction in genetic diversity also limits the cultivation range of a crop into environments that are more extreme than those in which it was domesticated, including “sustainable” agricultural systems with reduced inputs of pesticides, water, and fertilizers (Warschefsky et al., 2014). Climate change and the consequent rise of extreme weather events has understandably shifted plant breeders' priority toward breeding for abiotic stress tolerance (Hussain, 2015). Wild progenitors and current relatives of crop plants possess high levels of genetic diversity, which underlie an expanded range of adaptive traits that may be of agricultural relevance, including tolerance to abiotic extremes, and reduced dependence on inputs (Warschefsky et al., 2014). The potential for genetic gains from use of crop wild relatives is well documented (Pimentel et al., 1997; Tanksley and McCouch, 1997; Maxted and Kell, 2009) and the fact that they are under‐used in crop improvement programs represents an untapped opportunity. Moreover, new advances in genomics, phenotyping, and computational approaches can be used to infer natural adaptations in situ and improve breeding programs (Warschefsky et al., 2014).

This review summarizes our current understanding of the impact of the water stresses flooding and drought, on the performance of cereal crops, focusing on rice, wheat, barley, and maize. We present the latest knowledge on resilience mechanisms garnered from investigations of wild relatives of these crops. We cite evidence to support crop wild relatives as an invaluable reservoir of tolerance traits that can be used to advance efforts to produce climate‐resilient crops and secure food production.

## DROUGHT STRESS

There is no unifying definition for drought in the agricultural context. Drought can be defined as a prolonged period with insufficient rainfall which consequently depletes soil water via transpiration or evaporation, making it inadequate for crop demands (Jaleel et al., 2009; Folger and Cody, 2014; Kebede et al., 2019). Various plant parameters influence drought‐induced plant responses, including drought severity and duration, plant genotype, growth conditions, and developmental stage (Kantar et al., 2011; Akpinar et al., 2012).

Drought stress can cause various morphological, physiological, biochemical, and molecular changes in both the below‐ground and above‐ground tissues of cereal plants. The water content being reduced, the plant experiences a diminution of leaf water potential and a turgor loss. Leaf curling, partial, or complete stomatal closure, decrease in cell enlargement and growth, and a decrease of internal CO_2_ causing a decrease of photosynthetic activity are some of the physiological changes upon drought (Ridley and Todd, 1966; Centritto et al., 2009; Pandey and Shukla, 2015; Dawood et al., 2019). These morpho/physiological alterations lead to a reduction of leaf area and leaf development (Rizza et al., 2004; Nezhadahmadi et al., 2013; Kadam et al., 2015), of growth rate and, consequently, a thickening of the roots (Budak et al., 2015; Kadam et al., 2015) leading to an overall reduction of plant growth. If the stress is too severe, photosynthesis can stop, perturbing general metabolic activities and ultimately leading to the death of the plant (Jaleel et al., 2008). In triticale, if drought occurs during the pre‐anthesis stage, it shortens the time to anthesis while its occurrence post anthesis reduces the period of grain filling (Estrada‐Campuzano et al., 2008). The major negative impact of drought stress on crop plants is the reduction in fresh and dry biomass production (Farooq et al., 2009), which affects grain number and grain size in wheat (Dickin and Wright, 2008), and grain yield in maize (Kamara et al., 2003; Monneveux et al., 2006).

To reduce the risk of yield losses and to develop more drought‐tolerant crops, we need to understand how plants integrate drought cues with growth and development. Drought tolerance is defined as the ability to sustain growth, flowering, and yields even in sub‐optimally hydrated soils (Farooq et al., 2009). A common response in plants coping with drought stress is stomatal closure. This stabilizes the cell turgor and permits continued cellular metabolism. However, since stomatal closure also impairs the photosynthetic rate, plants must constantly adjust stomatal conductance to maintain a balance between sufficient CO_2_ uptake and water loss. Plants must then permanently sense water deficit. Abscisic acid (ABA) has been described to play a crucial role in stress signaling during drought at different levels, including transcriptional changes and promoting stomatal closure (Shinozaki and Yamaguchi‐Shinozaki, 2007; Cramer et al., 2011; Sreenivasulu et al., 2012).

A drought‐tolerant plant can maintain cellular and physiological function, including growth and seed/fruit production, under conditions of drought stress (Chaves et al., 2003). For a plant to be able to continue its cellular and physiological functions during drought, it needs to use the available water efficiently. Water use efficiency (WUE) is the ratio between the carbon gain to the water use/loss (Leakey et al., 2019). Improved WUE is an important trait in plant breeding (Prasch and Sonnewald, 2015). In the context of drought, this can be enabled by the constitutive generation of deep roots, the generation of “stay‐green” cultivars, enhanced proline accumulation (via *P5CR* gene) which promotes cellular protection and reactive oxygen species (ROS) scavenging, and the use of accumulated stem reserves for grain filling (Blum, 2016). Drought tolerance can also be enhanced by the accumulation of late embryogenesis abundant (LEA) proteins (Jones and Rawson, 1979). These hydrophilic proteins offer protection against desiccation damage through various means including antioxidant activity, and membrane and protein stabilization (Chen et al., 2019).

### Drought stress in barley, wheat, and wild relatives

**Figure 1:**
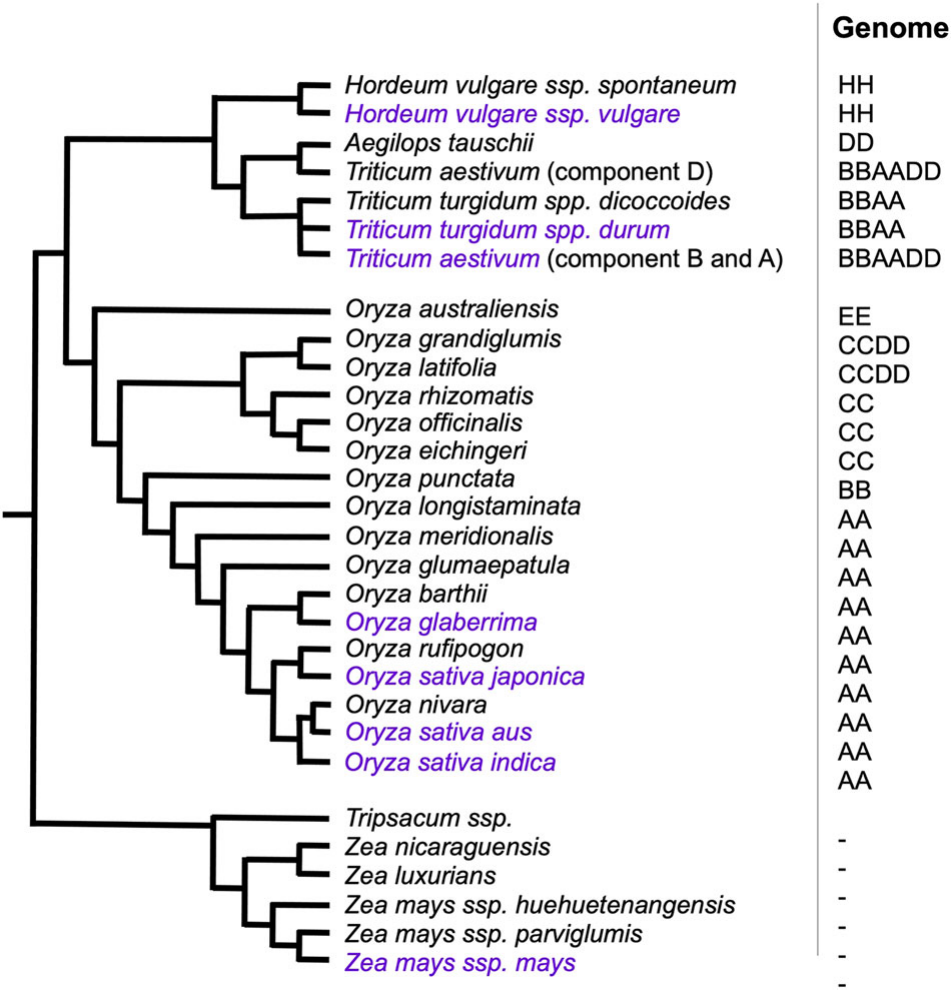
Species tree showing the relatedness between the crop and their wild relative species mentioned in this review Lines represent the relatedness between species. Distances and lengths of the lines are not representative of evolutionary time. Cultivated species are highlighted in purple. Column next to the tree depicts genome composition of the species. Tree built with information from Petersen et al. (2006), Wang et al. (2011), Stein et al. (2018), Haas et al. (2019), Shenton et al. (2020), Chen et al. (2021), Fu (2021), and plants.ensembl.org.

Common wheat (*T. aestivum* L.) and barley (*Hordeum vulgare* L.) are among the most important cereal crops with a more drought‐tolerant profile compared to the other cereal crops (Sallam et al., 2019), making them the perfect model system to highlight here. They, together with rye (*Secale cereale*), are members of the Tribe *Triticeae* of the *Poaceae* family and therefore share many genetic and biochemical characteristics (Chňapek et al., 2012; Figure 1).

**Figure 2:**
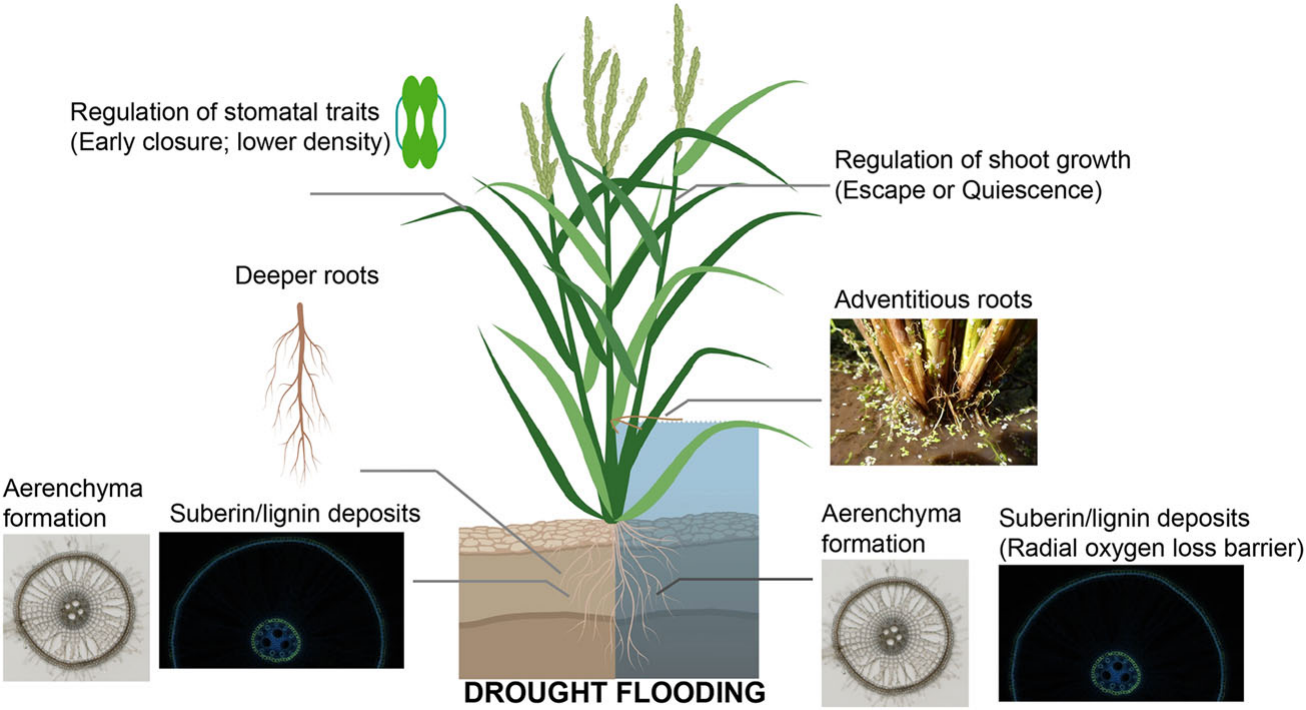
Overview of morphoanatomical traits that improve plant performance during drought (left) and flooding (right) Morphoanatomical traits in drought‐tolerant plants include early closure of the stomata or reduced stomatal density (“Regulation of stomata traits”) to improve water use efficiency (WUE) and a change in root architecture such as an increase in primary root length (“Deeper roots”) to facilitate water uptake from deeper soil layers. In flooded plants, the stress of reduced oxygen availability is eased via traits such as formation of “Adventitious roots” typically formed in the better aerated upper water layers and enriched with aerenchyma for better internal aeration. Depending on the flooding regime, plants also resort to adjusting shoot growth (“Regulation of shoot growth”) to either escape the water via accelerated shoot elongation or conserve energy using a quiescent strategy involving inhibition of shoot growth. Traits beneficial for coping with both stresses include: “Aerenchyma formation” and “Suberin/lignin deposits.” Aerenchyma are air‐filled spaces that can form throughout the plant and serve to enhance internal aeration in flooded plants. They can also be advantageous during drought since it reduces the number of energy‐demanding cells thus potentially favoring energy utilization toward improved WUE. Suberin and lignin deposits form apoplastic barriers in the root and are beneficial during flooding by preventing radial oxygen loss and during drought by preventing water loss. Images: Aerenchyma‐root cross section rice (Nipponbare); apoplastic barrier ‐ rice root cross section stained for suberin (Nipponbare); adventitious roots image (wild rice *Oryza glumaepatula*;). Image credits: Dr. Shiono Katsuhiro (Fukui Prefectural University, Japan). The plant image was created with Biorender (www.biorender.com).

Transpiration through stomata is the main reason for water losses from plants (Jasechko et al., 2013; Coenders‐Gerrits et al., 2014; Schlesinger and Jasechko, 2014). Several studies in dicotyledon plants, including *Arabidopsis*, have demonstrated that drought tolerance and WUE can be improved, if the stomatal frequency in leaves is reduced (Yu et al., 2008; Lawson et al., 2014; Hepworth et al., 2015). The molecular development of stomata in grasses is quite like *Arabidopsis* despite the discrepancies in their shape and patterning (Vatén and Bergmann, 2012). In monocots, stomata are formed via asymmetrical cell division from an epidermal cell at the base of the leaf to create an immature guard mother cell (GMC) (Boetsch et al., 1995). GMC maturation is controlled via the basic helix‐loop‐helix transcription factors (TFs), SPEECHLESS called HvMUTE (ortholog to *Arabidopsis* TF MUTE; Liu et al., 2009). Asymmetrical division of mature GMCs form pairs of the dumbbell‐shaped guard cells which is regulated by the epidermal patterning factor HvEPF1 (ortholog to *Arabidopsis* EPF family; Simmons and Bergmann, 2016). HvEPF1 belongs to a family of secreted signaling peptides, which are negative regulators of stomatal density (International Barley Genome Sequencing Consortium, 2012; Hughes et al., 2017). Barley lines overexpressing *HvEPF1* are significantly more drought tolerant (Hughes et al., 2017). This was associated with a higher WUE without a reduction in the grain yield upon water deficit due to a reduced stomatal density (Figure 2). The authors hypothesized that reduced rates of stomatal conductance and subsequently decreased water loss due to reduced stomatal density, allowed the available plant resources to be allocated to seed propagation and above‐ground biomass. Xeric genotypes of barley which were able to survive in arid conditions also displayed low stomatal conductance (Guoxiong et al., 2002). These findings suggest that manipulating stomatal frequency can be a promising avenue for breeders to optimize cereal yields in arid environmental conditions. Considerable knowledge for understanding the mechanisms of enhanced WUE and thus drought resistance can be gained from the wild relatives of wheat and barley (Nevo and Chen, 2010). *Triticum dicoccoides* (wild emmer wheat; wild relative of wheat; Figure 1) exhibited high productivity and WUE in arid conditions compared to cultivated durum wheat (*Triticum durum* or *Triticum turgidum* spp. *durum*) (Peleg et al., 2005; Table 1). A cross between wild emmer and durum wheat resulted in a tetraploid wheat population of 152 recombinant inbred lines (RILs). These lines were evaluated in the field for their productivity and drought tolerance traits. The analysis showed that out of the 110 quantitative trait loci (QTL) related to phenology, physiology, and productivity, only 20 QTL were associated with productivity (and related traits) upon water‐limited conditions. Interestingly, two loci (2B and 7B) could confer higher grain yield with the 7B loci being the most drought‐tolerant in wild emmer wheat alleles (Peleg et al., 2005). Similar findings were reported in double haploid lines deriving from wild barley (*Hordeum spontaneum* or *H. vulgare* spp. *spontaneum*; wild relative of barley; Figure 1) crossed with the cultivar Barke (*H. vulgare* spp. *vulgare*). Six out of 81 QTL contributed from wild *H. spontaneum* showed a positive effect on yield production upon drought, with two specific loci in chromosome 2H and 5H eliciting the highest yield increase (Nevo and Chen, 2010; Table 1).

**Table 1:** Mechanisms of water stress tolerance identified in cereal crop wild relatives

Crop	CWR	Stress	Gene/QTL location	Tolerance mechanism	Reference
*Hordeum vulgare* spp.	*H. vulgare* spp. *spontaneum*	Drought	*Hsdr4*	Drought tolerance in seedling stage	Supronova et al., 2007
		Drought	*EIBI1*	Drought tolerance in cuticle formation stage	Chen et al., 2004
		Drought	QTL (Chr 1H to 7H)	Better performance of tiller number and root dry weight, root volume and root length	Naz et al., 2014
		Drought	QTL (Chr 2H to 5H)	Higher grain yield upon drought	Nevo and Chen, 2010
*Triticum aestivum*	*Triticum dicoccoides*	Drought	QTLs (Chr 2B to 7B)	Higher grain yield and WUE upon drought	Peleg et al., 2005
	*Aegilops tauschii*	Drought	*Wrab13* (*LEA* gene)	Salt and dehydration tolerance	Iehisa and Takumi, 2012
*Oryza sativa*	*O. australiensis*, *O. glaberrima*, *O. longistaminata*, *O. meridionalis*	Drought	––	Thick leaves high mesophyll conductance to CO_2_ diffusion	Scafaro et al., 2011, Giuliani et al., 2013
	*O. rufipogon* genotype Dongxiang	Drought	––	Higher proline and soluble sugar accumulation	Zhang et al., 2014
	*O. rufipogon O. longistaminata*	Drought	––	Greater membrane stability higher stomatal conductance under drought	Neelam et al., 2018
	*O. glaberrima*	Drought	QTL ypp9.1, located at locus RM208 (Chr 2)	Early stomatal closure at the start of the drought period	Bimpong et al., 2011a, 2011b
	*O. rufipogon*	Drought	*OsDREB1F*	High relative water content, low leaf rolling score	Wang et al., 2008, Singh et al., 2015
	*O. nivara*				
	*O. glumaepatula*	Submergence	*SK2* and *SK2‐like* genes	Internodal elongation under deep‐water conditions	Hattori et al., 2009
	*O. grandiglumis*	Submergence	––	Quiescence strategy at the seedling stage elongation at a later stage	Okishio et al., 2015
*Zea mays* spp. *mays*	*Z. mays* ssp. *huehuetenangensis*	Waterlogging	QTL (Chr 3, 7, and 8)	Adventitious root formation improving access to oxygen	Mano et al., 2005b
	*Z. nicaraguensis*	Waterlogging	QTL (Chr 1, 5, and 8)	Constitutive root aerenchyma formation enhancing oxygen availability in the root and transport from shoot to root	Mano et al., 2006b, 2007, Mano and Omori, 2008
	*Z. luxurians*	Waterlogging/non‐stressed conditions			Ray et al., 1999, Mano et al., 2006b
	*Tripsacum dactyloides*	Non‐stressed conditions			Ray et al., 1999
	*Z. nicaraguensis*	Waterlogging/low oxygen in hydroponic conditions	QTL located at locus *RBF1*, (Chr 3 of teosinte)	Prevents oxygen loss from roots to the surrounding soil and facilitates oxygen diffusion inside adventitious and lateral roots	Watannabe et al., 2017, Pedersen et al., 2021
	*Z. nicaraguensis*	Waterlogging	QTL Qft‐rd4.07‐4.11 (Chr 4)	Reduces leaf injury and root damage	Mano and Omori, 2013, Mano and Nakazono, 2021

CWR, crop wild relative; QTL, quantitative trait loci; WUE, water use efficiency.

Several genes from ancestors of wheat and barley have been identified to play a key role in drought tolerance. *Hsdr4* and *EIBI1* genes are associated with drought tolerance in seedling and cuticle formation stage in wild barley (Chen et al., 2004; Suprunova et al., 2007; Table 1), while the dehydrin genes *Dhn1* and *Dhn6* are drought‐responsive genes from wild barley (Suprunova et al., 2004). Transcriptomic analysis of the drought response of a tolerant wild wheat genotype identified several genes and TFs associated with various hormones, in particular ABA (Ergen et al., 2009). The genome of *Aegilops tauschii* (wild relative of wheat; Figure 1) contains several drought‐responsive genes, making it an invaluable resource for improving wheat drought tolerance (Jones et al., 2013). Synthetic wheat lines and *A. tauschii* showed differential ABA responsiveness, based on the expression of ABA‐induced TF *WABI5* and the downstream *LEA* genes (*Wrab17*, *Wdhn13*, and *Wrab13*) (Iehisa and Takumi, 2012; Table 1). The study found that the highly ABA‐responsive lines had higher expression of *Wrab13* causing salt and dehydration tolerance. Proteomic analysis in another wild wheat relative *Kengyilia thoroldiana*, demonstrated several ABA signaling proteins to be involved including mitogen‐activated protein kinase 6 (MPK6), ABA 8′‐hydroxylase and dehydrin 3 (Yang et al., 2015).

Roots are very important in the context of drought since they can move in search of water (Hawes et al., 2000). Roots absorb water and nutrients from the soil which is later transported to the rest of the plant (Zingaretti et al., 2013). Roots are also the first organ to experience drought stress (Shimazaki et al., 2005; Kreszies et al., 2018). Monocot root systems are organized in concentric circles of different cell types, with the epidermis being the outermost cell layer in contact with the soil. Going inward there is the exodermis, different layers of cortical cells and the endodermis that surrounds the pericycle (Atkinson et al., 2014). There are two pathways of water transportation in the root. The “apoplastic pathway” which can be blocked by suberin lamellae and Casparian bands in endodermal and exodermal cell walls and the “cell‐to‐cell pathway” that is regulated via aquaporins (Barberon and Geldner, 2014; Kreszies et al., 2019). Suberin lamellae contain suberin (Bernards, 2002; Graça, 2015), while the Casparian bands contain mostly lignin (Naseer et al., 2012; Lupoi et al., 2015). The formation of suberin is dynamic and can be enhanced upon biotic and abiotic conditions (Lanoue et al., 2010). Barley seminal roots (*H. vulgare* L. spp. *vulgare* cv Scarlett) showed an increased suberin deposition in the endodermal cells during osmotic stress (Kreszies et al., 2018). This was also confirmed by RNA‐seq in which high expression of suberin biosynthesis genes (such as *HORVU3Hr1G085020*, *HORVU1Hr1G042910*) was observed during osmotic stress‐induced suberin deposition in barley seminal roots. Suberin enhancement upon water deficit could be an effective acclimation mechanism against drought by sealing the central vascular cylinder and preventing passive water loss (Figure 2). These mechanisms can be important breeding targets to avoid water losses from the root and improve WUE upon water‐deficit conditions.

Root to shoot communication is also very valid to understand drought‐tolerance mechanisms. A recent study using computational modeling showed that both shoots and roots can regulate stomatal responses to drought. A water potential decrease around the roots lead to faster stomatal closure than increased transpiration (Carminati and Javaux, 2020). Moreover, it has been reported that in barley there is a positive correlation between the root system size and grain yield, and the tiller number is closely related to the root number per individual plant (Hockett, 1986; Chloupek et al., 2010). Wild barley introgression lines (including genome‐wide introgressions of wild barley ISR42‐8 (*H. vulgare* ssp. *Spontaneum*) in cultivar Scarlett (*H. vulgare* ssp. *Vulgare*) background) displayed a better performance of tiller number and root dry weight, root volume and root length compared to their cultivar background Scarlett under drought. Fifteen chromosomal regions, with exotic QTL alleles, across chromosomes 1H to 7H were found to affect one or more of the previously mentioned traits (Naz et al., 2014; Table 1). These findings indicate that the transfer of QTL alleles from wild relatives to modern cultivars can improve drought tolerance. However, backcrossing with the wild parent will be necessary to refine the favorable QTL regions and gene position for any future breeding approaches.

### Drought stress on rice and wild relatives

Rice (genus *Oryza)* is composed of 24 species (two domesticated and 22 wild) distributed globally. It contains 10 distinct genome types: six diploid (2*N* = 24: AA, BB, CC, EE, FF, GG) and four polyploid (2*N* = 48: BBCC, CCDD, HHJJ, and HHKK) (Kim et al., 2008; Huang et al., 2012; Liu et al., 2015; Stein et al., 2018; Figure 1). The two domesticated rice are the Asian rice *Oryza sativa*, and the African rice *Oryza glaberrima* (Figure 1). Wild rice possesses many valuable features which might have been unintentionally lost during domestication, including tolerance to different biotic and abiotic stressors thus making them a good genetic resource for rice improvement (Sun et al., 2001; Li et al., 2009; Huang et al., 2012; Yu et al., 2021). Rice production is highly water dependent, with approximately 31% of the world's total rice production area under rainfed agriculture (Wassmann et al., 2009; Dixit et al., 2014). As a result, rice farming is particularly vulnerable to drought.

The AA genome species *Oryza meridionalis, O. longistaminata, O. glaberrima*, and *O. barthii* are good candidates for investigating drought tolerance based on their distribution in temperature and moisture extremes, as well as both forms of *O. punctata*, showing a high plasticity across moisture extremes (Atwell et al., 2014). Furthermore, *O. australiensis, O. glaberrima, O. longistaminata*, and *O. meridionalis* have been documented to possess thick leaves and have a high mesophyll conductance to CO_2_ diffusion. This alludes to drought tolerance since these traits can be associated with a higher WUE (Scafaro et al., 2011; Giuliani et al., 2013; Table 1). In the CC genome group, *O. rhizomatis* is confined and well adapted to highly disturbed habitats in dry zones in Sri Lanka. Its small, fragmented populations have evolved a high level of genetic diversity, making it a good wild species candidate for exploring drought‐tolerant traits (Bautista et al., 2006). Among the wild relatives tested in fields, some accessions of *O. glaberrima* were promising donors for improving drought tolerance in *O. sativa* breeding programs (Ndjiondjop et al., 2010). The *O. rufipogon* genotype Dongxiang is also a wild relative of interest as introgressed lines displayed enhanced drought tolerance in *O. sativa*. The drought tolerance of one introgressed line even surpassed the *O. sativa* parent possessing a higher survival rate, along with higher proline and soluble sugar accumulation (Zhang et al., 2006; Table 1). Feng et al. (2012) also tested drought tolerance of eight accessions of *O. rufipogon* and confirmed the higher tolerance of these accessions to drought. Remarkably one of the tested *O. officinalis* (CC genome) accession even performed better under drought conditions than in control conditions.

In a field test of 1630 accessions of wild species rice germplasm (AA, CC, BBCC and CCDD) under drought conditions, seven accessions from *O. rufipogon*, four from *O. longistaminata* and one each from *O. officinalis* and *Oryza latifolia* were identified as drought tolerant (Neelam et al., 2018; Table 1). A greater membrane stability and higher stomatal conductance was observed among *O. rufipogon* and *O. longistaminata* accessions under water deficit, compared to *O. sativa*.

Traits could also be introgressed from the African *O. glaberrima* drought‐tolerant rice to the Asian *O. sativa* (Wambugu et al., 2019). Some accessions of African rice have the capacity to retain more transpirable water via early stomatal closure at the start of the drought period (Figure 2) (Bimpong et al., 2011a). This feature, coupled to the fact that African rice usually matures earlier, can be a tolerance strategy involving the avoidance of severe drought periods. Indeed, introgression lines derived from a cross between *O. glaberrima* and *O. sativa* had higher yields under drought conditions than the *O. sativa* parent (Bimpong et al., 2011b; Table 1). About half of the beneficial alleles in the novel drought‐related QTL were derived from African rice. Different QTL were associated with improved yield under drought conditions in fields, with one for grain yield per plant (ypp9.1) located at locus RM208 (Chr 2) being new. This QTL positively affected yield under stress, accounting for 22% of the genetic variation. Shaibu et al. (2018) also evaluated about 2 000 accessions of African rice for drought tolerance and found that some accessions had even higher yields under drought conditions than the CG14 *O. glaberrima* drought‐tolerant reference. These germplasms thus represent a vast reservoir of possibilities for breeding drought‐tolerant species.

The gene *OsDREB1F*, a known drought stress‐responsive TF (Wang et al., 2008; Singh et al., 2015; Table 1), is associated with higher tolerance in several *O. sativa, O. rufipogon*, and *O. nivara* genotypes from India, linked to a high relative water content and a low leaf rolling score. Another TF family known to enhance tolerance to abiotic stresses is the NAC TF family. So far, 151 NAC TF genes have been identified in rice (Puranik et al., 2012). The overexpression of the *SNAC1* gene significantly enhanced drought tolerance in transgenic rice (22%–34% higher seed setting rate than the control) at the reproductive stage in the field under severe drought stress conditions (Hu et al., 2006).

As mentioned before, root‐related traits are of particular interest toward generating drought‐tolerant rice cultivars. Historically, breeding and domestication have focused on above‐ground traits potentially leading to genotypes with marginalized root systems. This might have resulted from the selection of germplasm with a better grain weight to whole plant biomass. While such reduced root system genotypes perform well under well‐irrigated conditions, there are serious drawbacks in arid soils. Root architecture can influence whole plant performance. Indeed, in the screen for drought tolerance across accessions of wild species rice germplasm (AA, CC, BBCC, and CCDD) (Neelam et al., 2018), the extent of leaf rolling and leaf drying showed a strong association with the root morphology of these accessions. Depending on the accession, the root length or the number of primary roots were increased under drought conditions compared to the response in the reference indica rice elite cultivar (Figure 2). The difference could also underlie different tolerance mechanisms. More vigorous or deeper rooting might in certain drought situations be beneficial for yields. Deeper rooting in Kinandang Patong, an upland cultivar from the Philippines, was associated with the *DEEPER ROOTING 1* (*DRO1*) QTL on chromosome 9 (Uga et al., 2013). Introduction of *DRO1* in shallow rooting cultivars significantly increased the maximum root depth, with a greater percentage of roots in deeper soil layers. This was linked to a stronger gravitropic effect in the roots. Importantly, there was no impact on the total root biomass or on the shoot architecture. In field experiments, DRO1 containing rice near isogenic lines (NILs) outperformed their controls despite water limitation, with effects most evident during severe drought. Dro1‐NIL plants had significantly higher photosynthetic capacity, deeper roots and elevated yields. The exploration of wild rice species that thrive in a range of arid environments is likely to reveal more loci like *DRO1* controlling beneficial root architectural traits.

## FLOODING STRESS

Most crops are sensitive to waterlogging (root submergence) and submergence (partial or complete submergence of aerial parts). Just a few days of flooding can seriously damage plants and will result in significant agricultural losses. The primary consequence of flooding is impaired gas diffusion between the plant and its environment. The gas diffusion rate in water is approximately 10 000 times slower than in air, resulting in limited delivery of O_2_ and CO_2_. This leads to an impairment of cellular respiration and of photosynthesis and consequently, an energy crisis that can ultimately kill the plant (Jackson, 1985; van Dongen and Licausi, 2015; Voesenek and Bailey‐Serres, 2015). Light availability can also be limited if plants are submerged in muddy water, further restricting photosynthesis. Impaired gas diffusion also causes accumulation of the gaseous hormone ethylene. While this is an important trigger of various adaptive responses (Sasidharan and Voesenek, 2015), long‐term ethylene build‐up can also be harmful (Stearns and Glick, 2003). In addition, oxygen deficiency in the soil causes reduction of oxidized compounds, which can be toxic to plants. The period following floodwater retreat presents another stressful scenario for flooded plants. The return to aerial conditions is associated with the excessive ROS accumulation leading to oxidative stress and drought‐like symptoms associated with malfunctioning roots (Yeung et al., 2019).

Plant responses to flooding include various morphological, metabolic and growth adjustments. These include alterations that facilitate hypoxia escape or when the flooding is too deep, hypoxia endurance. Escape traits typically permit avoidance of oxygen deficiency by improving internal aeration and enhancing underwater gas exchange. These include root traits such as the formation of a barrier against radial oxygen loss (ROL) in roots. This ROL barrier prevents the diffusion of oxygen to the surrounding anoxic soil, thanks to a deposition of lignin and suberin in the outer root cell layer (Colmer, 2003; Colmer et al., 2019; Pedersen et al., 2021). Such layers might also prevent the influx of toxic compounds from the soil into the plant. Often the primary roots are replaced by shoot‐borne adventitious roots that can be rich in aerenchyma. Aerenchyma formation can occur in roots, stems, and leaves (Figure 2). These interconnected gas‐filled spaces improve internal aeration by offering a low resistance pathway for the diffusion of air to flooded tissues from plant parts still above the water. Aerenchyma can be formed constitutively or in response to waterlogging or drought. This process is induced directly by the intercellular presence of ROS and of ethylene (Ni et al., 2019). Some species have hydrophobic leaves facilitating formation of gas films underwater which enhance gas exchange and photosynthesis (Pedersen et al., 2009). An upward movement of leaves (hyponasty) and shoot elongation can facilitate an escape response of plants from flood waters (Hattori et al., 2008; Ayano et al., 2014; Voesenek and Bailey‐Serres, 2015; Yamauchi et al., 2018; Pucciariello, 2020). In contrast, some plant species display a quiescence strategy upon submergence, meaning that shoot elongation is inhibited, and energy expenditure is limited until the water level decreases (Xu et al., 2006; Akman et al., 2012).

In addition to these different morphological and anatomical mechanisms, plants can also adjust their metabolism to oxygen‐limited conditions. This involves a switch to anaerobic metabolism to continue to produce energy (adenosine triphosphate (ATP)) or permits the anaerobic mobilization of starch to germinate underwater (Kretzschmar et al., 2015). This allows many wetland species to germinate anaerobically or to tolerate hypoxia during prolonged submergence. In plants that are tolerant to flooding, genes encoding alcohol dehydrogenase (ADH) and aldehyde dehydrogenase are increasingly expressed during submergence (Fukao et al., 2003). These enzymes use ethanol and acetaldehyde, respectively, to generate energy through fermentation.

Flooding‐tolerant species thus possess a suite of morphological, physiological, and metabolic adaptations to cope with the compound stress inflicted by flooding.

### Flooding stress on rice and wild relatives

Rice has traditionally been grown in flooded environments owing to its relative tolerance to waterlogged conditions (Mackill et al., 2012). Indeed, modern rice cultivars derive from aquatic ancestral landraces. However, prolonged, and deep flooding can also kill rice plants. Most rice varieties die within 14 days of complete submergence. Only a few can withstand more than 2 weeks of complete submergence (Xu et al., 2006). Being farmed in low lying flood‐prone areas, rice cultivation has always been vulnerable to flooding. But the heightened threat of flooding due to climate change makes the need for development of flood‐tolerant rice cultivars more urgent. In rice farming areas, flooding patterns can vary widely, with each requiring a different survival mechanism. Flooding regimes can vary from long‐term partial submergence (stagnant flooding (SF)) to complete plant submergence of short (flash floods) or long (deep‐water flood) duration.

Flash flood‐tolerant rice typically shows growth retardation when submerged and are highly tolerant of post‐submergence stress. An example is the Indian flood‐tolerant variety FR13A derived from a traditional landrace (Dhalputtia, an aus variety). The observed high submergence tolerance of FR13A was found to be conferred by the (*Submergence1*) *SUB1* QTL on chromosome 9 (Xu et al., 2006). The *Sub1* locus is composed of a cluster of three ethylene response factor (*ERF*) genes located in tandem, named *Sub1A*, *Sub1B*, and *Sub1C*. Tolerant genotypes possess the tolerant *Sub1* haplotype *Sub1A‐1/Sub1C‐1* (Singh et al., 2010). The dampening of underwater shoot growth imposed by *Sub1A* is mediated by an accumulation of the gibberellic acid (GA) signaling repressors Slender rice (SLR1) and SLR1 Like‐1 (SLRL1) (Fukao and Bailey‐Serres, 2008). The resulting repression of GA action subsequently results in restricted leaf and internode elongation. The downregulation of energetically expensive growth is accompanied by a reduced expression of genes involved in carbohydrate metabolism such as alpha amylases and sucrose synthases. In contrast, expression of genes encoding fermentation enzymes is activated. Thus, *Sub1A* directs a general energy conservation strategy while prioritizing core hypoxia acclimation responses (Bashar et al., 2019). This conservative strategy is especially beneficial for plants following desubmergence. *Sub1A* containing varieties show superior recovery associated with a better energy balance and higher drought and oxidative stress tolerance (Xu et al., 2006; Fukao et al., 2011; Tamang and Fukao, 2015). The introduction of the *Sub1A* locus into several varieties of high‐yielding rice allowed them to tolerate complete submergence for 2 weeks. For example, the introgression of the *Sub1* loci into the Indian variety Swarna (Swarna‐Sub1), resulted in high submergence tolerance in field trials without any negative influence on yield, plant height, harvest index and grain quality (Xu et al., 2006). Although *SUB1A‐1* derives from the aus sub‐group of indica rice (Xu et al., 2006), alleles have been also found in other *Oryza* species such as *O. nivara* and *O. rufipogon* accessions, also belonging to the A‐genome group. Other species such as *O. rhizomatis* and *O. eichingeri*, belonging to the C‐genome group, do not possess the *Sub1A‐1* allele but still are tolerant to flooding alluding to *Sub1A*‐independent mechanisms. Indeed, submergence tolerance is a common feature of many wild *Oryza* species that grow in wet habitats but do not possess *Sub1A*. Deeper investigation of such species is warranted to uncover novel tolerance mechanisms and loci (Niroula et al., 2012).

Many delta and river basin regions around the world experience long‐lasting floods several meters deep. In such flooding scenarios, deep‐water rice varieties thrive. They do so by utilizing an escape strategy where energy and carbohydrates are invested in stem elongation. This allows deep‐water rice to emerge above the water surface and continue normal oxygen uptake facilitated by aerenchyma. Deep‐water rice varieties, although not high yielding, are an important crop in flood‐prone regions of countries like Thailand, Bangladesh and Cambodia. The genetic and molecular mechanisms underlying the spectacular growth responses have been extensively studied (Kuroha et al., 2017, 2018). A QTL mapping approach identified three QTLs that explained the submergence‐induced elongation in the deep‐water rice cv C9285, a japonica varietal group from Bangladesh (Wang et al., 2013). The major QTL on chromosome 12 contained the genes *SNORKEL 1* (*SK1*) and *SNORKEL 2* (*SK2*), also members of the group VII ERFs. Ethylene accumulation in submerged internodes upregulates *SK1* and *SK2* gene expression. These TFs subsequently trigger downstream responses culminating in GA biosynthesis and stimulation of internodal elongation.

Coleoptile elongation is also seen as one of the major contributors to flooding tolerance in plants (Kato‐Noguchi and Morokuma, 2007). This means that flood‐tolerant rice varieties that show such an escape strategy will be more tolerant to flooding than tolerant rice varieties that show a quiescence strategy during early development. However, this elongation costs a lot of energy. To have a sustainable energy trade‐off, rice plants first produce the coleoptile before the radicle, and will invest less, or nothing at all, in root formation (Fox et al., 1994). *Oryza glumaepatula*, a wild rice species growing in areas along the Amazon River in South America that are flooded during the rainy season, responds to deep‐water conditions (Hattori et al., 2009; Table 1). *O. glumaepatula* possesses *SK2* and *SK2‐like* genes, but not *SK1*, suggesting that the former is important for the deep‐water response. Because some wild rice species possess *SK* genes, these genes may be of interest for breeding for flooding tolerance.

The knowledge gathered so far clearly demonstrates how a fine‐tuned growth response, in collaboration with morphological and metabolic adjustments, are required for various flooding regimes. Interestingly, ethylene‐mediated activation of group VII ERFs (*SUB1A, SK1, 2*) appears as a common theme for both escape and quiescent responses. The associated tolerance loci are clearly successful for triggering the appropriate coping mechanisms in the case of a short or prolonged flood. However, a major challenge remains in breeding for SF tolerance. Superior performance in SF conditions is associated with a more intermediate elongation capacity and high tillering (Kuanar et al., 2017). Not surprisingly, most SUB1 varieties do not perform well during SF (Singh et al., 2011; Vergara et al., 2014; Kato et al., 2019; Kuanar et al., 2019). So far, only Singh et al. (2017) identified several QTL associated with yield and agronomic traits under SF conditions, in a F7 RIL mapping population derived from a cross of the popular high‐yielding Indonesian variety Ciherang‐Sub1 (IR09F436) and an International Rice Research Institute submergence and SF tolerant breeding line IR10F365. This underscores the importance of further exploring the vast repertoire of *Oryza* species adapted to diverse hydrological regimes. For example, *O. grandiglumis*, a tetraploid species with CCDD genome lacking both the *SUB1A* alleles and the *SK1* and *SK2* genes, grows in Amazonian flood plains and is tolerant to both gradual and full submergence. This species shows a quiescence strategy at the seedling stage when fully submerged but can also elongate at a later stage when the water level gradually rises. It is hypothesized that the enhanced internodal elongation of submerged *O. grandiglumis* plants is not triggered by ethylene accumulated during submergence but by the moist surroundings provided by submersion (Okishio et al., 2015; Table 1). While the mechanisms underlying this mutable growth strategy remain unresolved (Okishio et al., 2014), it clearly makes a strong case for exploring wild germplasms.

### Flooding stress on maize and wild relatives

Modern maize (*Z. mays* subsp. *mays*) diverged from its wild relative teosinte (*Z. mays* subsp. *parviglumis*) about 9 000 years ago (Matsuoka et al., 2002; Piperno et al., 2009; Stitzer and Ross Ibarra, 2018). This single domestication event favored vegetative and reproductive traits for high yield and cultivation in a wide range of geographic zones (Doebley et al., 2006; Stitzer and Ross Ibarra, 2018). Nevertheless, regions prone to flooding remain challenging for maize production since the compound nature of this stress alter adaptive responses at different levels (Drew, 1997; Subbaiah and Sachs, 2003; Perata et al., 2011). Moreover, dynamic responses to excess water throughout maize development hinder comparative assessments of tolerance among different genotypes and crop improvement by traditional methods (Zaidi et al., 2004; Liu et al., 2013; Kaur et al., 2019, 2020).

Flooding effects on maize are highly dependent on developmental stage, genetic background, and frequency and duration of flooding (Zaidi et al., 2004; Kaur et al., 2019). Young plants, up to V6 (six visible leaf collars) are most susceptible to water damage, especially if the whole plant is submerged (Singh and Ghildyal, 1980). Since maize shoot apical meristem prior to V6 is near or below soil surface, the reduced gas diffusion and low‐light conditions inherent to flooded soils restrict photosynthesis and respiration in shoots and roots (Fukao et al., 2019). Submerged plants at this stage thus starve quickly and die within 24–48 h (Singh and Ghildyal, 1980). Moreover, even when plants survive, severe root damage during waterlogging can induce drought stress if the diminished root system cannot replenish transpirational losses after water recedes (Fukao et al., 2011; McDaniel et al., 2016). Conversely, established plants at the vegetative state (V7 to V10) become more tolerant to overly wet conditions and can endure longer periods of standing water without significant effects on biomass (Zaidi et al., 2004; Ren et al., 2014, 2016). However, waterlogging near silking and tasseling reduces yield by hampering pollination and seed set (Zaidi et al., 2004; Mangani et al., 2018).

Soil properties such as temperature, composition, and microbiome also alter the extent of flooding injury in distinct ways. For instance, temperature modulates metabolic activity of plant tissues and microorganisms around the rhizosphere. Time‐course analyses of pre‐emergent seedlings and established plants indicated that cool temperatures (10 °C) reduced early variations to hypoxia/flooding among maize genotypes including inbreds, hybrids, and wild relatives (Lemke‐Keyes and Sachs, 1989). Moreover, survival was maintained at 100% three times longer at 10 °C compared to 2%–35% at 25 °C–27 °C (Fausey and McDonald, 1985; Lemke‐Keyes and Sachs, 1989). Furthermore, previously flooded plants can become deficient in essential nutrients if these run off during flooding or are present in forms that are toxic or unavailable for root uptake (Wenkert et al., 1981; Lizaso et al., 2001; Kaur et al., 2020). In addition, overly wet conditions favor growth of harmful pathogens making plants more susceptible to diseases. Any of these factors can hamper maize recovery even after a plant has survived the hypoxic phase of waterlogging. Since direct and indirect factors alter plant responses to flooding, efforts to improve crop resilience could benefit from selecting traits for tolerance to any of these effects. Understanding individual and collective impacts of flooding in domesticated and wild species is thus central to generate and identify varieties resistant to water extremes.

Molecular understanding of flooding effects in maize includes well‐documented changes in transcription, protein, and metabolic levels (Sachs et al., 1980, 1996; Chen et al., 2014; Block et al., 2020; Hofmann et al., 2020; Sanclemente et al., 2021). Many of these responses have been studied in the context of low oxygen (hypoxia), which predominantly affects roots during waterlogging. Early work in maize identified the first set of plant anaerobic proteins (ANPs) essential for hypoxia acclimation (Sachs et al., 1980). These proteins mediate major pathways associated with sugar metabolism, glycolysis, and fermentation (Subbaiah and Sachs, 2003). Since their expression and activity is essential for anaerobic cell survival, ANP genes are often considered major targets for re‐engineering flood tolerance. For example, *Pyruvate decarboxylase* (*Pdc*) genes code for an enzyme that mediates ethanolic fermentation and improves post‐hypoxic survival when over‐expressed in *Arabidopsis* and rice (Quimio et al., 2000; Ismond et al., 2003). In maize, *Pdc* and other fermentation‐associated genes are highly accumulated in tolerant lines Mo18W and M162W after 24–72 h of submergence compared to sensitive lines B73 and B97 (Campbell et al., 2015). These observations highlight the potential of targeting these paths to improve flooding resilience in maize. However, messenger RNA accumulation for ANPs and activity does not always correlate with long‐term flooding tolerance, despite their central role in rapid acclimation responses to hypoxia. For instance, the enzyme ADH, a widely‐used marker of low oxygen metabolism, mediates ATP production through the conversion of acetaldehyde to ethanol (Andrews et al., 1994; van Dongen and Licausi, 2015). Maize plants lacking functional *Adh* genes are less tolerant to low oxygen and typically die within a few hours of hypoxia (Wignarajah and Greenway, 1976; Good and Crosby, 1989; Loreti et al., 2005). Nevertheless, *Adh* overexpression did not confer enhanced long‐term tolerance to flooding in several plant species (Ismond et al., 2003). Furthermore, genetic variation for seedling tolerance to low oxygen in maize is not associated to levels of ADH activity (Lemke‐Keyes and Sachs, 1989). These two contrasting examples highlight the importance of characterizing and understanding the physiological significance of flooding stress components for future breeding applications. Although several genes for low oxygen acclimation have been identified in maize, only a few studies have directly tested their potential to improve tolerance. Furthermore, oxygen restriction is considered the main source of injury during flooding. However, the compound nature of this stress implies that it is regulated by a complex network of genetic and molecular factors that can limit genetic improvement through single‐gene approaches.

Genetic analyses of maize accessions subjected to either low oxygen or flooding have identified tolerant germplasms and the genetic basis of associated traits. For instance, crosses between exotic accessions of Mexican maize with high tolerance for anoxia and sensitive inbreds Mo20W and B73Ht showed that loci for tolerance were dominant and had single segregation (Lemke‐Keyes and Sachs, 1989). Furthermore, studies using RILs have characterized several QTL important for flood resistance in maize. Initial assessment of an F2 population derived from a cross between inbreds F1649 (flood‐tolerant) and H84 (sensitive) revealed the first genomic regions associated with shoot survival and dry matter located in chromosome 1 (Mano et al., 2006a). Subsequent analyses of waterlogged‐tolerant Hz32 and the sensitive K12 indicated that two major QTL in chromosomes 4 and 9 were associated with enhanced flood survival (Qiu et al., 2007). Moreover, secondary QTL in chromosomes 1, 2, 3, 6, 7, and 10 also contributed to the response. Consistently, Zaidi et al. (2015) also showed that QTL on chromosomes 1, 3, 5, 7, and 10 explained up to 30% of the variation in grain yield between the waterlogged‐tolerant line CAWL46‐3‐1 and the sensitive line CML311‐2‐1‐2‐3. These regions carry genes for TFs, ROS scavengers, and anaerobic metabolism. Additional QTL for secondary traits such as brace roots and contributions from each parental line were identified and explained 3%–14% of the waterlogging phenotypes (Zaidi et al., 2015). In addition, Campbell et al. (2015) found a QTL named *Subtol6* on chromosome 6. This region contains genes for Hemoglobin 2 (Hb2) and the DNA‐binding protein RAV1 that explained 22% of the variation between Mo18W (tolerant) and B73 (sensitive). From these two genes, *Hemoglobin* is a promising candidate since overexpression of plant and bacterial isoforms have been shown to increase maize survival during anoxia, waterlogging and submergence (Dordas et al., 2004; Zhao et al., 2008; Du et al., 2016).

Large variations in flood tolerance within the *Zea* genus and other panicoid grasses provide an invaluable resource for genetic improvement. Prominent examples of maize relatives with known tolerance to water extremes include teosinte and *Tripsacum* spp. These species are cross‐compatible with maize and can be used to transfer genetic regions for important agricultural traits (Sahoo et al., 2021). Introgressions between maize and wild relatives are also advantageous since they can be used to partially enhance genetic diversity lost during domestication (Sahoo et al., 2021).

Two close‐related species of teosinte, *Zea nicaraguensis* and *Zea luxurians*, have emerged as promising sources of germplasm to improve flooding tolerance in maize (Figure 1). *Z. nicaraguesis* is typically found in lowland areas of the northwest coast of Nicaragua that remain flooded for most of the year (Iltis and Benz, 2000), while *Z. luxurians* grows in regions of high precipitation in Oaxaca, Mexico (Sánchez González et al., 2018; Gonzalez‐Segovia et al., 2019). Tolerance to excess water and root zone hypoxia in these species have been associated with the capacity to grow adventitious roots, form root aerenchyma (Figure 2), presence of ROL barrier and tolerance to toxic compounds commonly found in reduced flooded soils (Mano and Omori, 2007; Mano and Nakazono, 2021). Introgressions of these traits into maize have been tested and shown to enhance survival of resulting hybrids in F1 and F2 populations (Mano and Nakazono, 2021). Initial studies by Mano et al. (2005a, 2005b) identified QTL associated with adventitious root formation (ARF) in two F2 populations derived from crossing maize inbred lines (ILs) B64 (low ARF) × teosinte (*Z. mays* spp *huehuetenangensis*) (high ARF) and B64 (low ARF) × Na4 (high ARF) (Figure 1). QTL for ARF were mapped to chromosomes 3, 7, and 8 with loci in chromosome 8 being consistent in both populations (Mano et al., 2005b). Further investigations indicated that both, B64 and Na4 as wells as *Z. luxurians* and *Z. nicaraguensis* could form root aerenchyma during flooding (Mano et al., 2006b). Although most elite maize lines do not form aerenchyma in aerated or non‐stress conditions, introgression of this trait in this crop may enhance its capacity to grow in environments with contrasting water regimes. Initial crosses between *Z. mays* × *Tripsacum dactyloides* and *Z. luxurians* × *Z. mays* showed formation of root aerenchyma in F1 hybrids creating new paths to improve root aeration under water extremes in this crop (Ray et al., 1999; Table 1). Loci for constitutive aerenchyma formation found in chromosomes 1, 5, and 8 explained up to 46.5% of the variation in the F2 population of maize B64 × *Z. nicaraguensis* (Mano et al., 2007). The potential for using these QTL for marker‐assisted breeding was verified in backcrossed populations of F1 hybrids of elite maize Mi29 × *Z. nicaraguensis* (Mano and Omori, 2008). Consistently, loci for aerenchyma formation were also found in chromosomes 1 and 5. Furthermore, maize seedlings of introgression lines with QTL for constitutive aerenchyma in the Mi29 background showed greater tolerance to low oxygen and flooding stress (Gong et al., 2019).

Similar Mi29 × *Z. nicaraguensis* crosses have been used to identify QTL for formation of ROL barrier and tolerance to reduced flooded soils. Characterization of Mi29 × *Z. nicaraguensis* hybrids and the NILs derived from these crosses (NIL468‐3) revealed that a locus named *RBF1* regulates ROL barrier formation in adventitious and lateral roots (Watanabe et al., 2017; Pedersen et al., 2021). Authors also found that *RBF1* derives from a segment of chromosome 3 of teosinte and that the associated gene(s) are dominant since phenotypes are observed in homozygous and heterozygous material (Watanabe et al., 2017). Initial assessment of Mi29 × *Z. nicaraguensis* hybrids in reduced soil conditions identified a tolerant line (IL#18) that has a large segment of chromosome 4 of teosinte. The QTL was named *Qft‐rd4.07‐4.11* (QTL for flooding tolerance under reducing soil conditions at bin 4.07–4.11 (Mano and Omori, 2013; Mano and Nakazono, 2021)). When this locus was introgressed into maize Mi47 and Na50, the resulting F1 hybrids showed enhanced flood tolerance (Mano and Omori, 2015). However, it was later observed that linkage drag hampered the potential of *Qft‐rd4.07‐4.11* in further breeding studies since a chromosome inversion abolished recombination in this locus (Mano and Omori, 2013). Genetic assessments are currently underway to generate and identify tolerant lines that carry chromosome segments of *Z. nicaraguensis* that confer tolerance to reduced soil conditions (Mano and Nakazono, 2021).

## CONCLUSION

Leverage of naturally occurring genetic diversity is crucial to generate crops resilient to changing environments. Examples presented here in grains indicate that relatives of modern cultivars represent a tremendous reservoir of naturally evolved traits for abiotic stress resistance (Figure 2). New methods and tools have expedited the exploration and utilization of this genetic diversity including speed breeding (Watson et al., 2018), gene editing (Chen et al., 2019), and large‐scale, automated phenotyping (Smith et al., 2021). Moreover, advances in sequencing technologies, assembly methods, and computational tools can now generate more robust, high‐quality genome sequences for discovery applications. For instance, end‐to‐end (chromosome) RNA sequencing paired with *de novo* genome assembly have facilitated the construction of (super)pangenomes that capture the whole range of genetic variation within a species or genus (Khan et al., 2020). Pangenomes for many crops including maize and rice are already available in public repositories and have been used to uncover novel sequences for traits of interest and structural variations (>50 bp) in genomic regions regulating these traits. For example, in rice, analysis of genome assemblies for multiple genotypes available at the RicePanGenome database (http://db.ncgr.ac.cn/RicePanGenome/) were recently used to analyze allelic variations of *Sub1* genes (Singh et al., 2020). In addition, authors there identified sources of flood tolerance in 18 cultivars that were not associated with *Sub1* genes, which could be used to develop superior lines for flood resistance (Singh et al., 2020). In maize construction of a pangenome using diverse ILs, large segments (>1 000 Kb) of chromosome rearrangements were identified, including insertions, deletions, and inversions in all 10 chromosomes. The largest of these rearrangements (~75.5 Mb) was found in chromosome 2 of only three of the 66 lines assembled. Using clustered regularly interspaced short palindromic repeats (CRISPR)/CRISPR‐associated protein 9 (Cas‐9), authors there were able to revert this region making it more accessible for recombination of important traits (Hirsch et al., 2014; Schwartz et al., 2020). Emergence of pangenomic studies offer several advantages over analyses based on individual reference genomes since they allow for more accurate single nucleotide polymorphism (SNP) imputation for haplotype prediction, SNP discovery, and control of recombination sites for breeding purposes (Della Coletta et al., 2021). CRISPR/Cas‐9 has emerged as the most commonly used technique for genome editing in cereals in recent years (Ansari et al., 2020). The availability of reference genomes for the major cereals have expedited the efficient use of this technology to improve various agronomic traits including those related to herbicide and/or biotic and abiotic resistance in rice, wheat, barley and maize. For more about the utilization of CRISPR/Cas technology in cereal crops, we direct readers to a recent detailed review (Matres et al., 2021). Collective use of these developments for crop improvement have significantly eased the identification and transfer of tolerance loci from wild relatives. Several QTL for traits of interest have been mapped and successfully transferred into crops. However, the genetics and molecular understanding of their regulation remains limited. Identification of genes associated with tolerant phenotypes is central to distinguish their roles in short‐ and long‐term survival. Moreover, characterization of these loci can help define specific genomic targets and methods to transfer these without the potential limitations of linkage drag often associated with the introgression of ancestral alleles.

Unfortunately, many crop wild relatives are at risk of extinction from habitat loss and fragmentation, changing land use, management practices, climate change, and introgression from agricultural relatives (Ford‐Lloyd et al., 2011). Thus, a high priority should be given for studying and conserving wild relatives of crops (crop ancestors and descendants) (Ellstrand et al., 2010; Warschefsky et al., 2014; Castañeda‐Álvarez et al., 2016; Dempewolf et al., 2017). Currently, plans for conservation (Heywood et al., 2007; Zhang et al., 2017) and sequencing of wild relative genomes (Dempewolf et al., 2014; Brozynska et al., 2016; Khan et al., 2020) are implemented in several programs including: the Millennium Seed Bank of the Royal Botanic Gardens, Kew (https://www.kew.org/wakehurst/whats-at-wakehurst/millennium-seed-bank), the Global Crop Diversity Trust (https://www.croptrust.org), the IPK's BRIDGE project for barley (“https://bridge.ipk-gatersleben.de” https://bridge.ipk-gatersleben.de), DivSeek (“https://divseekintl.org/” https://divseekintl.org/), The Crop Wild Relatives project (“https://www.cwrdiversity.org/” https://www.cwrdiversity.org/) on wild relatives, and the International Weed Genomics Consortium (IWGC; https://www.weedgenomics.org/) on weeds (Maroli et al., 2018; Ravet et al., 2018; Sharma et al., 2021).

In addition to crop wild relatives, another potential source of novel traits for crop improvement are weeds. While weeds are unwanted in the field, their ability to invade and colonize a range of environments demonstrates impressive environmental adaptability. They also abound in interesting traits that could be used for new agronomic challenges. For example, studies on *Echinochloa* spp. growing in paddy fields have been useful to identify flooding tolerance traits to introgress in rice, including improved anaerobic germination, photosynthesis, and post‐submergence survival (Bouhache and Bayer, 1993; Ismail et al., 2012; Covshoff et al., 2016; Khedr et al., 2017). We direct readers to Sharma et al. (2021), for an excellent insight on the relevance of weeds in uncovering novel stress resilience mechanisms and traits.

Finally, while most studies focus on one stress, several adaptive traits can serve to enhance survival through different and even opposite environmental stresses. For example, the implications of breeding for anatomical modifications for flooding tolerance extend to drought as well. This is the case of aerenchyma formation in cortical cells, which can help balance root metabolism with plant demands for water and nutrients when water is limited (Zhu et al., 2010). The identification and characterization of such multi‐stress‐related traits is essential considering that in the field stresses often occur either sequentially or simultaneously (Rivero et al., 2021).

**Glossary:**

**Description of selected terms as defined for the scope of this review**

1. **Weeds:** Weeds are plant species growing in human‐controlled settings (fields, gardens, lawns, parks, roadsides, etc.), that are unwanted for varied reasons including negative impact on crop yield or the aesthetics of a place.
2. **Crop (wild) relatives (CWRs)**: “A CWR is a plant that is more or less closely related to a crop and to which it may contribute genetic material, but unlike the crop species has not been domesticated; they are also likely to be the progenitors or direct ancestors of crops”. A CWR can be a weed or a crop, as most if not all species are potential gene donors to a crop. From Heywood et al. (2007).
3. **Natural landraces**: “A landrace is a dynamic population(s) of a cultivated plant that has historical origin, distinct identity, and lacks formal crop improvement, as well as often being genetically diverse, locally adapted and associated with traditional farming systems.” From Villa et al. (2005).
4. **Flooding**: “A general term referring to excessively wet conditions, that is where excess water replaces gas‐spaces surrounding roots and/or shoots.” Waterlogging refers to a flooding of only the root system. Submergence refers to a partial or complete submersion of the shoot. From Sasidharan et al. (2017).
5. **Drought**: Drought stress is experienced by plants when water in the soil becomes insufficient to meet all functional requirements for a normal growth and development.
6. **Water stresses**: Stressful plant growth conditions caused by either excess (flooding) or limitation (drought) of water.
7. **Acclimation/adaptation**: “Adaptation” is the genetic process by which a population changes to accommodate to a “new” environment, whereas “acclimation” is the physiological changes the plant makes to minimize the effects of the stress.
8. **Avoidance**: Stress‐induced morphological adaptations that result in a reduction of the water loss in the case of drought, or prevention of oxygen loss in the case of flooding.

## CONFLICTS OF INTEREST

The authors declare they have no conflicts of interest associated with this work.
